# Otoferlin Is Required for Proper Synapse Maturation and for Maintenance of Inner and Outer Hair Cells in Mouse Models for DFNB9

**DOI:** 10.3389/fncel.2021.677543

**Published:** 2021-07-14

**Authors:** Ursula Stalmann, Albert Justin Franke, Hanan Al-Moyed, Nicola Strenzke, Ellen Reisinger

**Affiliations:** ^1^Auditory Systems Physiology Group, Department of Otolaryngology and Institute for Auditory Neuroscience, SFB 889 project A06, University Medical Center Göttingen, Göttingen, Germany; ^2^Molecular Biology of Cochlear Neurotransmission Group, Department of Otolaryngology, University Medical Center Göttingen, Göttingen, Germany; ^3^Gene Therapy for Hearing Impairment Group, Department of Otolaryngology, Head and Neck Surgery, University of Tübingen Medical Center, Tübingen, Germany

**Keywords:** inner hair cells, outer hair cells, synapse maturation, degeneration, otoacoustic emissions, hearing loss, otoferlin, spiral ganglion neurons

## Abstract

Deficiency of otoferlin causes profound prelingual deafness in humans and animal models. Here, we closely analyzed developmental deficits and degenerative mechanisms in *Otof* knock-out (*Otof*^–/–^) mice over the course of 48 weeks. We found otoferlin to be required for proper synapse development in the immature rodent cochlea: In absence of otoferlin, synaptic pruning was delayed, and postsynaptic boutons appeared enlarged at 2 weeks of age. At postnatal day 14 (P14), we found on average ∼15 synapses per inner hair cell (IHC) in *Otof*^–/–^ cochleae as well as in wild-type controls. Further on, the number of synapses in *Otof*^–/–^ IHCs was reduced to ∼7 at 8 weeks of age and to ∼6 at 48 weeks of age. In the same period, the number of spiral ganglion neurons (SGNs) declined in *Otof*^–/–^ animals. Importantly, we found an age-progressive loss of IHCs to an overall number of 75% of wildtype IHCs. The IHC loss more prominently but not exclusively affected the basal aspects of the cochlea. For outer hair cells (OHCs), we observed slightly accelerated age-dependent degeneration from base to apex. This was associated with a progressive decay in DPOAE amplitudes for high frequency stimuli, which could first be observed at the age of 24 weeks in *Otof*^–/–^ mice. Our data will help to plan and predict the outcome of a gene therapy applied at various ages of DFNB9 patients.

## Introduction

Mutations in the gene *OTOF* encoding for the protein otoferlin cause hearing impairment with autosomal recessive inheritance, DFNB9 (Yasunaga et al., 1999). To date, more than 200 pathogenic mutations have been identified in the *OTOF* gene (Vona et al., 2020). While most mutations lead to profound deafness, a few non-truncating mutations result in mild to moderate progressive or temperature sensitive forms of hearing loss (reviewed in Vona et al., 2020).

The deficiency in otoferlin causes a defect in synaptic transmission from auditory sensory cells, the inner hair cells (IHCs), to the SGNs (Roux et al., 2006; Pangrsic et al., 2010; Strenzke et al., 2016; Michalski et al., 2017). Initially, OHCs, the active elements of the cochlear amplifier, are intact. This can be readily assessed by recordings of otoacoustic emissions (OAE). Due to the presence of OAEs but the absence of signal transmission in the auditory pathway, this form of hearing impairment was classified as “auditory neuropathy,” and since identification of the primary defect at the level of IHC synapses more precisely as “auditory synaptopathy.”

The current treatment of choice for patients with mutations in *OTOF* and profound hearing loss is cochlear implantation (CI). This device bypasses the affected IHC synapses by directly activating SGNs electrically. Indeed, the outcome of CI in early-treated DFNB9 is excellent (reviewed in Zheng and Liu, 2020). Recent experimental studies aim toward the development of a gene therapy to restore natural hearing in DFNB9 by transducing IHCs with the cDNA of otoferlin using adeno-associated viruses (AAVs). As the length of the otoferlin coding sequence exceeds the cargo capacity of AAVs, either an overload viral vector or a combination of two viruses (dual-AAV) were injected into the cochlea, which successfully rescued hearing function in *Otof*^–/–^ mice (Akil et al., 2019; Al-Moyed et al., 2019; Rankovic et al., 2020). Since overloading AAVs results in a mixture of truncated AAV genomes (Dong et al., 2010; Lai et al., 2010; Wu et al., 2010), this technique is rather difficult to be translated to human application. In contrast, clinical trials for dual-AAV treatment of human DFNB9 are currently being prepared, promoted by three different companies (Reisinger, 2020). DFNB9 seems to be an ideal candidate for gene therapy, since the pre-and postnatal development of the inner ear sensory epithelium and the morphology of IHC synapses judged by EM appeared initially normal (Roux et al., 2006). The same study unraveled a reduction in the number of intact ribbon-type synapses to 50% in *Otof*^–/–^ IHCs by immunofluorescence microscopy (Roux et al., 2006). More recent immunohistochemical studies found indications for an abnormal development of IHC synapses (Al-Moyed et al., 2019). As each IHC ribbon synapse is normally contacted by a single SGN, such a reduction in ribbon synapse numbers would be expected to be accompanied or followed by a decay of spiral ganglion neurons (SGNs), adversely affecting the outcome of treatments by CI or gene therapy.

In addition to a reduction in the number of afferent synapses, several studies revealed that OHC function deteriorates over time in people affected from *OTOF* mutations (Starr et al., 1996; Rodríguez-Ballesteros et al., 2003, 2008; Rouillon et al., 2006; Kitao et al., 2019). In about one third of the patients, OAEs vanished already within the first 2 years of life, and of the remainders, most lost OAEs by the age of 20 years (Rodríguez-Ballesteros et al., 2003, 2008; Kitao et al., 2019). While this loss of OHC function does not matter for cochlear implantation, it would be a great obstacle for the gene therapy, since a deficiency of OHC function would cause a sensitivity loss of up to 50–60 dB hearing level (HL).

To date, it is unclear why OAEs vanish. Kitao et al. (2019) proposed that expression of otoferlin in immature OHCs of normal hearing individuals might render those cells less susceptible to noise at later maturational stages. This hypothesis is based on the observation of otoferlin expression in immature OHCs in mice, which was found to decrease in the second postnatal week down to a level where it is not unambiguously detectable by immunohistochemistry or functional tests any more (Roux et al., 2006; Beurg et al., 2010). How the expression of otoferlin in immature OHCs might change the properties of mature OHCs to protect these from environmental factors such as noise remains to be determined. Nevertheless, a potential low-level expression of otoferlin in mature OHCs, if present, might play a role for prevention of OAE loss. This would be of significance for the design of the gene therapy, since, in this case, targeting not only IHCs but also OHCs would be required to restore hearing life-long. Another hypothesis argues that efferent down-regulation of OHC activity is missing in DFNB9 patients, which potentially leads to higher susceptibility to noise (Starr et al., 1996; Vona et al., 2020). Accordingly, the use of hearing aids was suspected to be causative or at least contribute to the loss of OAEs (Starr et al., 1996; Rouillon et al., 2006). Indeed, a longitudinal study in auditory neuropathy patients found indications supporting this hypothesis, though it was not conclusive if the use of hearing aids was really causative for the OAE decay (Kitao et al., 2019).

Here, we studied the development and degeneration of hair cells, synapses and SGNs in *Otof*^–/–^ mice from birth to 48 weeks of age. Our data unravels developmental deficiencies and degenerative processes in the inner ear in absence of otoferlin, which will be important for consulting patients prior to a gene therapy, and for planning the time point and refining the design of the gene therapy for DFNB9.

## Materials and Methods

### Animals, Animal Welfare and Handling

Animal handling and experiments complied with national animal care guidelines and were approved by the board for animal welfare of the University Medical Center Göttingen and the animal welfare office of the state of Lower Saxony, Germany.

**FIGURE 1:**
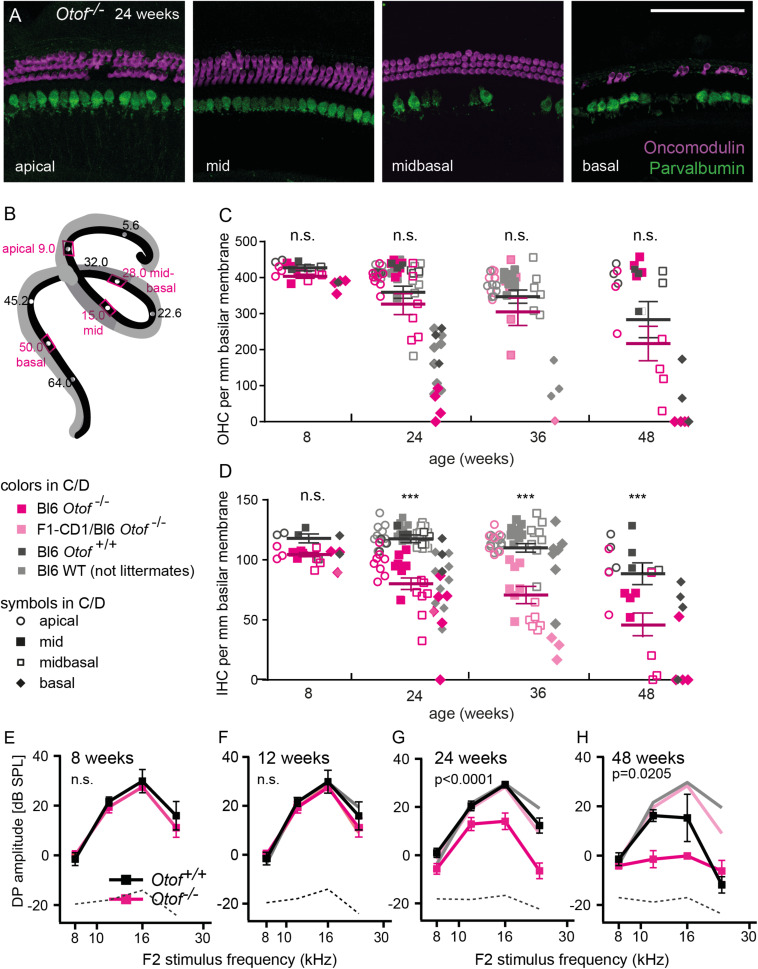
Inner and outer hair cell degeneration in absence of otoferlin. **(A)** Maximum projections of confocal micrographs of immunolabelled *Otof*^–/–^ whole mount organs of Corti in four regions of the cochlea with IHCs labeled for parvalbumin (green) and OHCs labeled for oncomodulin (magenta). Representative example of an *Otof*^–/–^ specimen at 24 weeks of age, scale bar: 100 μm. **(B)** schematic drawing of the mouse organ of Corti, with the regions analyzed in this study indicated in magenta. **(C)** number of OHCs per mm basilar membrane at 8, 24, 36, and 48 weeks of age in *Otof*^+^*^/^*^+^ (gray, mean ± SEM black) and *Otof*^–/–^ (magenta, mean ± SEM dark magenta) mice. *Otof*^–/–^ mice at 36 weeks of age were of mixed background (F1-CD1xC57BL6), all other mice of C57BL/6 background. Across all age groups and tonotopic regions, the number of OHCs in *Otof*^–/–^ is reduced to 88% of *Otof*^+^*^/^*^+^ counts (*p* = 0.0705, 2way ANOVA followed by Sidac test). **(D)** number of IHCs per mm basilar membrane indicated a strong decay of the number of IHCs in the absence of otoferlin (reduction to 69% of WT, *p* < 0.0001, 2way ANOVA). *** indicate age groups in which the reduction in IHC numbers across tonotopic regions was significant (*p* < = 0.0001, *post hoc* Sidac test after 2way ANOVA). E-H, Amplitude of the 2f1-f2 distortion product across four different stimulation frequencies (60 dB sound pressure level) at the age of 8 weeks **(E)**, 12 weeks **(F)**, 24 weeks **(G)**, and 48 weeks **(H)**. In panels **(F–H)**, the values obtained at 8 weeks are plotted in light colors for comparison. Note that both mouse lines are of C57BL/6 background, explaining the decline of DPOAE amplitudes in the wild-type controls at 48 weeks of age. The amplitude reduction in *Otof*^–/–^ compared to *Otof*^+^*^/^*^+^ is significant at 24 (*p* < 0.0001, *n* = 5/6) and 48 weeks (*p* = 0.0205, *n* = 4 each) of age but not at 8 (*n* = 6 each) and 12 weeks (*n* = 5/3) (2way ANOVA for genotype effect across frequencies). Dotted line: noise floor.

*Otof*^–/–^ mice (Reisinger et al., 2011) were backcrossed onto C57BL/6N or CD1 background for at least 5 generations. Littermate *Otof*^+^*^/^*^+^ mice were used as controls. For hair cell counts, we used C57BL/6N-*Otof*^–/–^ mice, *Otof*^–/–^ mice derived from homozygous matings of CD1 *Otof*^–/–^ females and C57BL/6N *Otof*^–/–^ males, and other *Otof*^+^*^/^*^+^ mice of C57BL/6N or C57BL6/J background, as indicated by color labeling in Figures 1C,D. Animals of both genders were used in the experiments. Mice were housed in social groups in individually ventilated cages in a specific pathogen-free facility with free access to water and food and a 12/12 h light/dark-cycle.

### Recording of Distortion Product Otoacoustic Emissions

Distortion product otoacoustic emissions (DPOAE) were recorded from mice anesthetized with ketamine (125 mg/kg) and xylazine (2.5 mg/kg) i.p. in a soundproof box as described previously (Jing et al., 2013). Briefly, a custom designed probe was inserted in the ear canal, containing a sensitive microphone (MKE-2, Sennheiser, Hannover, Germany) and silicone tubes connected to two speakers (MF1-S, Tucker Davis Technologies, Ft Lauderdale, FL, United States). Primary stimuli f1 and f2 were presented over 16 s at a frequency ratio f2 = 1.2xf1 and intensity ratio f1 = f2 + 10 with f1 frequencies of 6–23 kHz, controlled by a custom Matlab routine and TDT system III hardware (Tucker Davis Technologies, Alachua, FL, United States). Recorded emissions were fast-Fourier transformed and amplitudes of DPOAE at 2xf1-f2 measured and averaged over both ears.

### Immunohistochemistry

Cochlear whole mounts of mice at the age of postnatal day 4 (P4) to 48 weeks were immunostained as previously described (Strenzke et al., 2016). The cochleae were perfused and fixed with 4% formaldehyde for 10–30 min or 45 min (Figure 3B) at 4°C. Cochleae of mice older than 4 weeks were decalcified in Morse’s solution (10% sodium citrate, 22.5% formic acid) for 15 min or in 0.5 M EDTA overnight before dissecting the whole organ of Corti including basal turns. For cryosections of the cochlea, samples were perfused and fixed in 4% formaldehyde for 30 min and decalcified in Morse’s solution for 2–24 h. Cochleae were kept in 20% sucrose until embedding and cutting into 20 μm sections (Cryotome 2800 Frigocut, Reichert-Jung, Austria).

The following antibodies were used: mouse anti-Ctbp2 (#612044, BD Bioscience, 1:50–1:200) for labelling synaptic ribbons, rabbit anti-Na/K-ATPase (SC28800, Santa-Cruz Biotech, 1:200) or rabbit anti-Shank1a (#RA19016, Neuromics, 1:300) for labelling postsynaptic boutons (Huang et al., 2012), guinea pig anti-parvalbumin (#195004, Synaptic Systems, 1:200) to label IHC and OHC as well as SGN cell bodies and afferent fibers, guinea pig anti-Vglut3 (#135204, Synaptic Systems, 1:300) to label IHCs, goat anti-calretinin (CG1, Swant, 1:300) to label IHC and OHC, mouse anti-neurofilament (N5389, Sigma, 1:400) to label afferent fibers and SGN cell bodies, mouse anti-MyoVI (#H00004646-M02, Novus Biological, 1:200) or rabbit anti-MyoVI (#25-6791, Proteus, 1:200) antibodies for labelling hair cells, and rabbit anti-oncomodulin (OMG4, Swant, 1:300–1:1,000) for labelling OHC.

The following secondary antibodies were used: DyLight 405 donkey anti-guinea pig (Jackson Immuno, 1:200), Alexa Fluor 568 IgG H + L goat anti-mouse, Alexa Fluor 633 IgG goat anti-rabbit (MoBiTec, 1:200), Alexa Fluor 488 goat anti-guinea pig, Alexa Fluor 488 goat anti-mouse IgG, IgA IgM (H + L), Alexa Fluor 647 donkey anti-rabbit, Alexa Fluor 568 donkey anti-goat, Alexa Fluor 488 donkey anti-mouse (Invitrogen, 1:200). Hoechst 34580 (1:1,000, H21486, Fisher scientific) or To-Pro-3 Iodide (642/661, 1:800, T3605, ThermoFisher) were used as nuclear stains.

**FIGURE 2:**
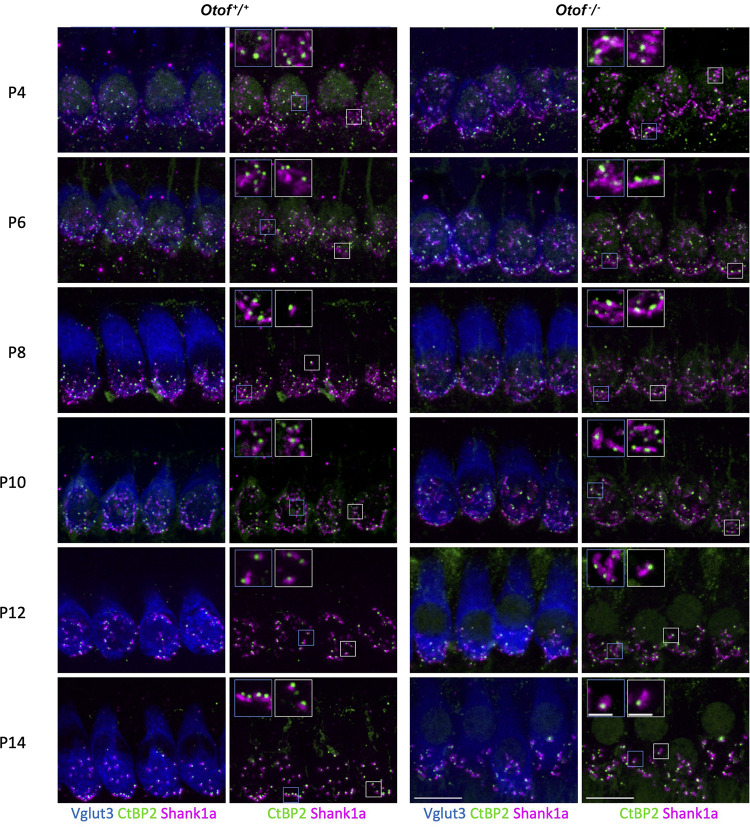
Immunohistochemistry of IHC synapses in the first two postnatal weeks. Two left columns: *Otof*^+^*^/^*^+^ controls, two right columns, *Otof*^–/–^ IHCs. In column 1 and 3, IHCs are visualized by Vglut3 immunostaining. Afferent synapses of *Otof*^+^*^/^*^+^ and *Otof*^–/–^ IHCs were immunolabelled for Ctbp2, which is identical to the B-domain of the ribbon component ribeye. Antibodies against Shank1a labeled postsynaptic boutons. Insets show higher-magnification views of representative synapses. Note that postsynapses of *Otof*^+^*^/^*^+^ controls become smaller during maturation, but not in *Otof*^–/–^ IHCs. Maximum projections of high-resolution confocal micrographs, scale bar 10 μm; scale bar of insets: 2 μm. P6 and P14 data are partially replotted from Al-Moyed et al. (2019).

**FIGURE 3:**
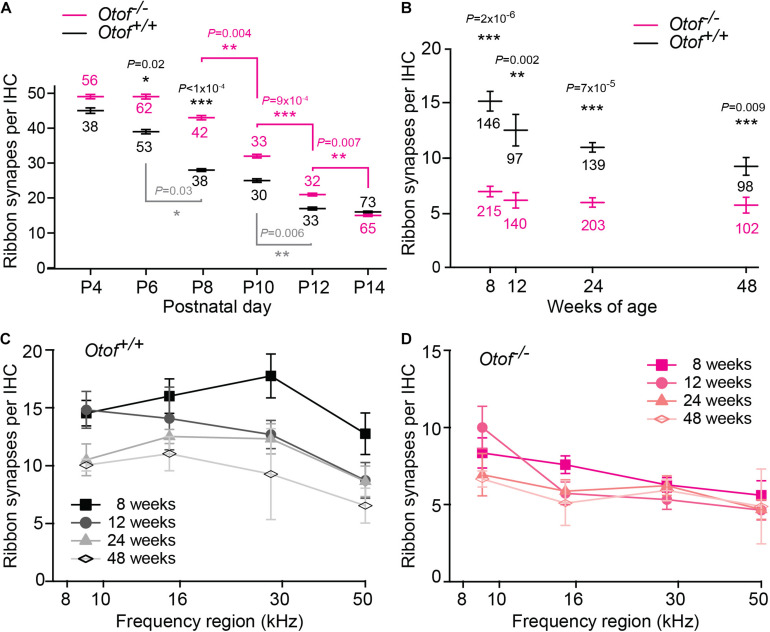
Decline of afferent synapses over time. **(A)** Quantification of afferent synapses (as juxtaposed signals of Ctbp2 and Shank1a) between postnatal day 4 and 14 (P4, P14) from immunolabeling as presented in Figure 2; *n* = 30–73 IHCs, as indicated in the figure, apical region with exception of the most apical ∼640 μm (P6 and P14 re-plotted from Al-Moyed et al., 2019). Only statistically different results between *Otof*^+^*^/^*^+^ and *Otof*^–/–^ IHCs are indicated (black *p* values), as well as significant changes during development within *Otof*^+^*^/^*^+^ (gray) and within *Otof*^–/–^ samples of different ages (magenta). **(B–D)** Numbers of synapses (as juxtaposed immunolabeling of Ctbp2 punctae with Na/K-ATPase labeled boutons); **(B)** between 8 and 48 weeks of age, averaged from synapse counts in the four regions of the cochlea indicated in Figure 1B (*Otof*^–/–^: 8 weeks: *N* = 7 cochleae; 12 weeks *N* = 5, 24 weeks: *N* = 8, 48 weeks: *N* = 4; wildtype: 8 weeks *N* = 5; 12 weeks: *N* = 3; 24 weeks: *N* = 4; 48 weeks: *N* = 4; IHC numbers are indicated in the figure). **(C,D)** Numbers of synapses per IHC plotted against cochlear frequency region for *Otof*^+^*^/^*^+^ **(C)** and *Otof*^–/–^ **(D)** IHCs. Statistics: **(A)** Kruskal-Wallis test followed by Dunn’s multiple comparison test; **(B)** F-test followed by Student’s *t*-test. ^∗^*p* < 0.05; ^∗∗^*p* < 0.01; ^∗∗∗^*p* < 0.001.

Confocal images were acquired using a laser scanning confocal microscope (Leica TCS SP5, Leica Microsystems GmbH, or QuadScan Super-Resolution Microsocope, Abberior Instruments, Göttingen, Germany) with a 10× air objective (low magnification), 20× or 40× oil immersion objective (medium magnification) or 63× oil (Figures 2 and 3A: 63× glycerol) immersion objectives for high magnification images. Z-stacks were acquired at 9, 15, 28, and 50 kHz frequency regions with high magnification for synaptic ribbon quantification and medium magnification for quantification of IHC, OHC, and SGN.

Images were processed and analyzed using ImageJ (NIH,^1^). In samples from adult mice, synaptic ribbons (immunolabelled for Ctbp2) and postsynapses (immunolabelled for Na/K-ATPase) were manually counted in 5–15 μm z-stacks (0.2 μm step size, 3.3× digital zoom) using ImageJ. In P4-P14 organs of Corti, synapses were counted as Ctbp2 spots adjacent to Shank1a immunolabels using the “Spots” tool in Imaris 7.6.5 (Bitplane Scientific Software). For quantification of synapse size at P14, we segmented the confocal stacks for Ctbp2- and Shank1a-positive spots, respectively, using the surface function in Imaris. The expected spot sizes were set to 0.55 μm for Ctbp2 and 0.8 μm for Shank2. Background subtraction and the rejection thresholds in terms of intensity, size, sphericity and quality of spots were optimized for each image to detect as many true synapses as possible in which Ctbp2 and Shank1a were juxtaposed with a maximal distance of 600 nm. We clearly acknowledge that the results of the volume calculations can easily be influenced by the subjectivity of these settings. We focused on correctly segmenting the Shank1a channel and are thus less sure about an observed smaller increase in Ctbp2 spot size by 20% (*Otof*^+^*^/^*^+^: 0.297 ± 0.006 μm^3^, *n* = 1,287; *Otof*^–/–^ 0.342 ± 0.006 μm^3^, *n* = 1,135; *p* < 0.0001).

Inner hair cell and OHC cell bodies were identified by nuclear and/or cell body staining (MyoVI/Parvalbumin and Hoechst) and manually counted along a defined length of basilar membrane measured along the row of IHC. Areas with dissection-induced damage to the preparation were carefully excluded from analysis. Hair cells with preserved cytoplasmic staining were counted as present even if they showed an irregular shape or position. For SGN quantification, we measured the area of the *canalis spiralis cochleae* and manually counted the density of SGN cell bodies (Hoechst staining co-immunolabeled with Parvalbumin).

### Statistics

All experiments were performed with at least three independent replicates. Data are plotted as mean ± standard error of the mean (s.e.m.) and graphics were designed using *Igor Pro* 6 (WaveMetrics). Statistical analysis was performed by means of *GraphPad Prism 7.03* (GraphPad Software) or *Excel* (Microsoft Office). The Brown-Forsythe test or the F-test were used to test for equal variance. Statistical tests and number of replicates are indicated in the figures or figure legends.

## Results

### Degeneration of Inner and Outer Hair Cells

In this study, we assessed the integrity of cochlear structures from birth to mid-aged *Otof*^–/–^ mice and wild-type C57BL/6 controls. We first quantified the number of inner and outer hair cells and of afferent synapses in four frequency regions, termed apical (best encoding frequencies around 9 kHz), middle (∼15 kHz), midbasal (∼28 kHz) and basal turns (∼50 kHz, Figures 1A,B; Müller et al., 2005). At the age of 8 weeks, *Otof*^–/–^ mice display a near-normal complement of a single row of IHCs and three rows of OHCs over the entire cochlea (Figures 1C,D). From 24 weeks of age on, a decay in numbers of IHCs and OHCs becomes apparent in *Otof*^–/–^ cochleae. While OHC numbers remain normal in the apical aspects of the cochlea in both genotypes, there is age-dependent loss of OHCs progressing from the base toward the middle of the cochlea, which is accelerated in *Otof*^–/–^mice compared to *Otof*^+^*^/^*^+^ controls. For example, in the midbasal and basal regions of *Otof*^–/–^mice at age 24 weeks, there was a reduction to 84 and 28% of *Otof*^+^*^/^*^+^ OHC numbers, respectively (Figure 1C). However, in the C57BL/6 mouse background strain, age-progressive hearing loss has previously been described and was attributed to the *Cdh23*^*ahl*^ allele (Kane et al., 2012; Mianné et al., 2016), explaining the almost complete loss of all OHCs in the 50 kHz regions by 48 weeks of age in both genotypes. As a result, the overall OHC numbers per age group were not significantly different between genotypes, despite the midbasal and basal OHC degeneration appeared more pronounced in *Otof*^–/–^mice compared to *Otof*^+^*^/^*^+^ controls.

In *Otof*^+^*^/^*^+^ mice, the row of IHCs was generally completely preserved, except for limited age-dependent degeneration of IHC in the basal turn. In contrast, scattered loss of IHCs was noted in *Otof*^–/–^ mice over all frequency regions (Figure 1D) from 24 weeks of age on. Like for OHC, the loss of IHC in mutants was more pronounced in the midbasal and basal regions, where at 24 weeks of age IHC numbers were reduced to 56 and 64% of *Otof*^+^*^/^*^+^ IHC counts, respectively, compared to 80 and 76% in the apical and middle aspects.

In summary, there is age-dependent loss of OHCs in control C57BL/6 *Otof*^+^*^/^*^+^ animals which is mostly restricted to the basal aspects of the cochlea and accompanied by only minimal loss of IHCs. In *Otof*^–/–^mice, IHC and OHC are initially present, but show a more pronounced age-dependent degeneration. While the loss of OHCs predominantly affects the basal and midbasal regions, IHCs degenerate also in the middle and apical turns to some extent, starting already at 6 months of age.

### Degeneration of the Cochlear Amplifier Over Time

Most patients with pathogenic *OTOF* mutations lose DPOAE within their first two decades of life. Here, we first tested if the loss of DPOAE is reproduced in the mouse models for DFNB9 (Figures 1E–H). At the age of 8 and 12 weeks, we found DPOAE to be of comparable amplitude in *Otof*^+^*^/^*^+^ and *Otof*^–/–^ mice. At the age of 24 weeks, DPOAEs were significantly reduced in *Otof*^–/–^ mice for stimulus frequencies (f2) of 12, 16 and 24 kHz compared to *Otof*^+^*^/^*^+^ mice, indicating a role for otoferlin in preserving OHCs and active cochlear amplification. Thereafter, DPOAE amplitudes declined further between 24 and 48 weeks of age for both genotypes. In *Otof*^+^*^/^*^+^ mice a mild reduction of DPOAE was observed for stimulus frequencies of 16 and 24 kHz, which can likely be attributed to the *Cdh23*^*ahl*^ allele in the C57BL/6 mouse background strain which is known to cause age-progressive high-frequency hearing loss (Kane et al., 2012; Mianné et al., 2016). A more severe DPOAE decline appeared in *Otof*^–/–^ mice between 12 and 24 kHz. Thus, the functional decline of cochlear amplification assessed as DPOAE was accelerated in absence of otoferlin and preceded the loss of OHCs. For both DPOAE loss and OHC degeneration, the high frequency regions were affected earlier and more severely than the low frequency regions of the cochlea.

### Maturation and Degeneration of Afferent IHC Synapses and SGNs in Absence of Otoferlin

A recent study indicated developmental deficits in *Otof*^–/–^ afferent synapses (Al-Moyed et al., 2019). This led us to count ribbon type synapses in frequent intervals during the maturation of IHCs shortly before the onset of hearing, which in rodents is at 2 weeks of age. In this period, the Shank1a-labeled postsynaptic boutons of wildtype IHC gradually become smaller and restricted to a small structure close to the ribbon, and extensive synaptic pruning occurs (Figures 2, 3A). Remarkably, postsynaptic boutons at *Otof*^–/–^ IHCs appeared morphologically different from age-matched wild-type boutons: The same immunostaining reveals that postsynaptic Shank1a-labeling remains in large patches throughout early postnatal development (Figure 2). At P14, Shank1a spot volume was increased by 50% (*Otof*^+^*^/^*^+^: 0.532 ± 0.012 μm^3^; *n* = 1,306 spots from 4 animals; *Otof*^–/–^ 0.802 ± 0.019 μm^3^, *n* = 1,068/*N* = 4; *p* < 0.0001; mean ± s.e.m, Wilcoxon rank sum test).

In two-day intervals, we determined the number of synapses in IHCs of the apical region, defined as Ctpb2 immunoreactive spots juxtaposed to Shank1a labeled postsynapses. In *Otof*^–/–^ IHCs of the apical region, we found an initially higher number of synapses between postnatal day 4 (P4) and P12, but a faster decay of ribbon synapse numbers in the absence of otoferlin compared to wild-type IHCs (Figures 2, 3A). Previous studies from our and other labs indicate that in the third and fourth postnatal weeks, ribbon synapse numbers in *Otof*^–/–^ IHCs decrease to 60% of the numbers in wild-type IHCs (Roux et al., 2006; Al-Moyed et al., 2019).

Together, our data reveal that the presence of otoferlin is required for synaptic maturation before the onset of hearing.

We next assessed the long-term effect of otoferlin-deficiency on synapse numbers in the four regions of the cochlea described in Figure 1B. Along the length of the cochlea, at 8 weeks of age, we found ∼7 synapses per *Otof*^–/–^ IHC. Between 12 and 48 weeks of age, we counted on average ∼6 synapses per *Otof*^–/–^ IHC, with no obvious decay in synapse numbers over time (Figure 3B). While synapse numbers in *Otof*^+^*^/^*^+^ IHCs for different frequency regions declined rather constantly over time (Figure 3C), no gross difference was apparent for the different turns of the cochlea regarding synapse numbers in *Otof*^–/–^ mice (Figure 3D).

**FIGURE 4:**
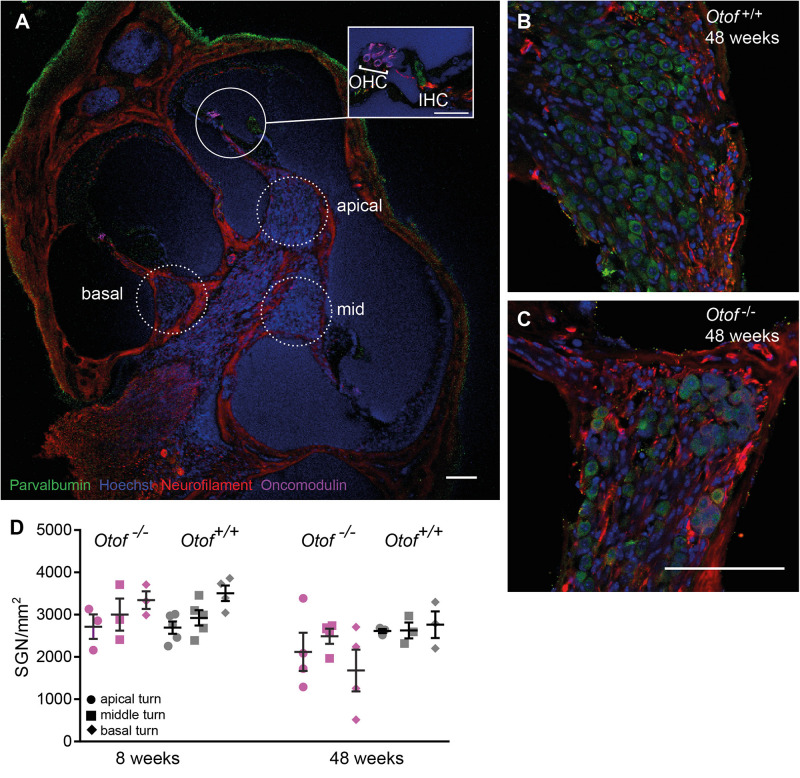
Number of SGN cell bodies at 8 and 48 weeks of age. Cochleae were cryosectioned (*N* = 3 to 5 cochleae, as indicated) and immunolabelled for parvalbumin, neurofilament, oncomodulin and nuclear stain (Hoechst). **(A)** Overview of a cochlear section (scale bar of inset: 30 μm), Note that since imaging settings were optimized for the brightest fluorescence, which is for parvalbumin in hair cells, the weaker expression of parvalbumin is hardly visible in SGNs. **(B,C)** Higher magnification view of Rosenthal’s canal in the apical cochlea, **(D)** Cell bodies were counted in 20 μm thick cryosections. SGN counts revealed a small but significant reduction in SGN numbers (*p* = 0.038, 2way ANOVA genotype difference across tonotopic regions) in *Otof*^–/–^ compared to *Otof*^+^*^/^*^+^ cochleae at age 48 weeks. Scale bar, 100 μm.

We next counted the number of spiral ganglion neurons (SGNs) at the age of 8 and 48 weeks (Figure 4). At 8 weeks of age, despite afferent boutons from these SGNs already being reduced to about half of wild-type numbers, the numbers of SGNs were comparable for *Otof^–/–^ and Otof*^+^*^/^*^+^ mice. Since studies of age-dependent and noise-induced synaptopathy revealed that a loss of SGN bodies occurs with a delay of several months or even years (Kujawa and Liberman, 2009, 2015; Sergeyenko et al., 2013), we counted SGN cell bodies again at the age of 48 weeks. Here, we found a slight but significant loss of SGN cell bodies at the age of 48 weeks in *Otof*^–/–^ mice to an overall of 79% of *Otof*^+^*^/^*^+^ SGN counts (*p* = 0.038, 2way ANOVA genotype difference across tonotopic regions). In terms of cochlear implantation and gene therapy, the long-term presence of SGNs is promising, since the remaining SGNs can be electrically stimulated and a re-growth of dendrites could potentially be initiated by endogenous or supplemented nerve growth factors.

## Discussion

In this study, we set out to analyze potential developmental defects and degenerative processes due to the absence of otoferlin in mice. For the first time, we unraveled a remarkable degeneration of IHCs between 8 and 48 weeks of age in *Otof*^–/–^ mice. Moreover, we found morphologically altered postsynapses and delayed synaptic pruning in the first two postnatal weeks of *Otof*^–/–^ IHC. The decay in OAEs in our mouse models over the time course studied well reflects the observed spontaneous loss of OAEs in human patients. The degradation of OAEs in *Otof*^–/–^ mice first affects the higher frequencies and proceeds to the frequencies of best hearing within months. It is followed by the loss of OHCs, IHCs and SGNs later on, all starting at the base of the cochlea. According to our data, it would be preferable to apply any gene therapeutical intervention as early as possible to yield maximal benefit and hopefully to prevent degradation of IHCs. However, the mouse model for DFNB9 analyzed here comprises a large genomic deletion of exons 14 and 15, causing a subsequent frameshift (Reisinger et al., 2011), and might not model the phenotypes of all DFNB9 patients accurately. Indeed, minimal auditory evoked responses were recorded in patients with premature STOP mutations, but not in patients with frameshift mutations (Santarelli et al., 2015; Vona et al., 2020). Thus, our *Otof*^–/–^ mouse line might reflect the phenotype of DFNB9 patients with frameshift mutations or large genomic deletions more closely than the one of DFNB9 patients with missense or point mutations.

### Developmental Deficit of Synaptic Maturation in *Otof*^–/–^ IHCs

Synaptic transmission is independent of otoferlin in immature IHCs up to postnatal day 3 (Beurg et al., 2010). From P4-5 on, hardly any Ca^2+^-triggered synaptic transmission can be recorded in *Otof*^–/–^ IHCs (Beurg et al., 2010). Up to postnatal day 6, spontaneous action potentials can be recorded from IHCs, giving rise to synaptic activation (Kros et al., 1998; Marcotti et al.,2003a,b). Thus, during early postnatal development, transsynaptic activity is supposed to be wild-type like in absence of otoferlin, but will be greatly diminished from P5 on in *Otof*^–/–^ mice. We found that synaptic maturation differs in absence and presence otoferlin from P6 on. Synaptic pruning in otoferlin-deficient IHCs started between P6 and P8, with a delay of 2 days compared to wild-type controls (Figure 3A). In *Otof*^+^*^/^*^+^ IHCs, synapse numbers remained mostly constant from P12 on, while they declined further in *Otof*^–/–^ IHCs. From the third and fourth postnatal weeks, ∼40–50% fewer synaptic contacts are present in *Otof*^–/–^ IHCs compared to littermate controls (Roux et al., 2006; Al-Moyed et al., 2019). A similar loss of approximately 40% of the ribbon synapses and delayed loss of SGNs has been described in *Vglut3*^–/–^ mice, in which IHC synaptic vesicles are not loaded with glutamate (Seal et al., 2008). Later, mRNA sequencing suggested that the low spontaneous rate/high threshold SGNs were missing in this mouse line (Shrestha et al., 2018). If glutamatergic signaling was the key factor for retaining low spontaneous rate/high threshold synaptic contacts, then presumably also in *Otof*^–/–^ cochleae this type of synapses would degenerate during development. Remarkably, 6 out of 7 synapses remaining at 8 weeks of age in each *Otof*^–/–^ IHC persist for at least 40 weeks. Possibly, the rare spontaneous synaptic events present in *Otof*^–/–^ mice, recorded as excitatory postsynaptic potentials in postsynaptic boutons (Pangrsic et al., 2010; Takago et al., 2019) support their maintenance, either by glutamatergic or by neurotrophic signaling. In contrast, a 90% reduction in voltage gated Ca^2+^ current in Ca_v_1.3 knockout mice not only reduced the number of synapses to ∼60% within the first 4 weeks of life, but also to a further decay down to ∼2 synapses per IHC at the age of 29 weeks (Nemzou et al., 2006).

Given the early maturational stage where an effect of otoferlin-deficiency on synapse development becomes apparent, a gene replacement would need to be applied even prenatally to ensure proper synaptic development. After successful gene substitution in rodents at P6-P7, the number of ∼7 synaptic contacts was unchanged, which was sufficient to elicit auditory responses after gene substitution (Al-Moyed et al., 2019). As a result, although in humans *OTOF*-gene therapy will take place at a time point when synapse numbers are presumably already reduced, a rescue of hearing function is likely to be successful.

In addition to the alteration in synapse numbers, postsynaptic boutons appear to be larger in absence of otoferlin. While Shank1a immunolabeling in P14 wild-type controls was confined to small postsynaptic patches, Shank1a immunofluorescence appeared expanded in *Otof*^–/–^ cochleae. Similarly, Kim et al. (2019) demonstrated enlarged postsynapses labeled for GluA3 in *Vglut3*^–/–^ mice, in which inner hair cells exocytose vesicles lacking glutamate. We presume that neurotransmitter release from presynaptic ribbon synapses regulates the size of the postsynaptic density.

Despite a reduction in synapse numbers in the third and fourth postnatal week, the number of SGNs was wild-type like in 8 week old *Otof*^–/–^ cochleae. Even at 48 weeks of age, only about half of the animals displayed a loss in SGN density (Figure 4D). Despite mRNA of otoferlin could be detected in (immature) SGNs (Yasunaga et al., 1999) and in brain (Yasunaga et al., 2000; Schug et al., 2006), our immunostainings of whole mount organs of Corti do not indicate that SGNs express significant amounts of otoferlin. Moreover, since cochlear implants that bridge only the IHC-SGN synapse have good long-term outcomes in DFNB9 patients, SGNs and neurons of the central auditory pathways seem not to functionally depend on otoferlin. We hypothesize that SGN cell bodies in *Otof*^–/–^ cochleae persist despite the loss of the IHC ribbon synapse for several months, similar to the long delay between a noise trauma leading to a 40% reduction in synapse numbers and the death of SGN cell bodies (Kujawa and Liberman, 2009, 2015; Sergeyenko et al., 2013). Due to the shorter life span, mice age much faster than humans do, such that we presume that SGNs might persist in DFNB9 patients for years even without therapeutic intervention. A re-growth of the dendrites, induced by application of nerve growth factors, is experimentally addressed in mouse models to initiate recovery of synaptic contacts (Chen et al., 2018; Hashimoto et al., 2019). More studies will be required to test if a re-establishment of afferent synapses could be achieved in *Otof*^–/–^ IHCs by a similar approach in the future.

### Degeneration of Inner and Outer Hair Cells and Decay of OAEs

Previous studies, some of which with longitudinal studies, found OAEs to degenerate in DFNB9 patients (Rodríguez-Ballesteros et al., 2003, 2008; Kitao et al., 2019). This is the first study addressing long-term effects in mouse models for DFNB9. Similar as in human patients, DPOAEs were wild-type like in the first weeks of life. In *Otof*^–/–^ mice, first signs of OAE decay were found at 24 weeks of age. At 48 weeks of age, a major deterioration of DPOAEs was observed for the 12 and 16 kHz region. In the basal turn, OAEs were strongly decreased also in *Otof*^+^*^/^*^+^ controls, most likely due to the *Cdh23*^*Ahl*^ allele leading to progressive hair cell degeneration (Kane et al., 2012; Mianné et al., 2016). While a more detailed study using mouse strains with a corrected *Cdh23*^*Ahl*^ would be useful to analyze OHC degeneration solely due to otoferlin deficiency, we demonstrate here that the loss of OHCs in *Otof*^–/–^ clearly precedes the one in *Otof*^+^*^/^*^+^ mice of the same background. At 24 weeks of age, wild-type *Otof*^+^*^/^*^+^ littermates and non-littermate controls of C57BL/6 background displayed consistently more OHCs around 28 and 50 kHz than *Otof*^–/–^ mice (Figure 1C). In addition, at 48 weeks of age, no loss of OHCs was apparent in the 28 kHz region of *Otof*^+^*^/^*^+^ mice, but ∼70% of OHCs were lost in *Otof*^–/–^ mice.

Importantly, our study is the first to elucidate the loss of IHCs in *Otof*^–/–^ mice. This loss was found along the length of the cochlea, with a shallow basal to apical gradient. In contrast to *Otof*^–/–^ cochleae, IHCs were mostly preserved in *Otof*^+^*^/^*^+^ controls even at 48 weeks of age, despite the presence of the *Cdh23*^*Ahl*^ allele. To date, it is unclear why *Otof*^–/–^ IHCs and OHCs degenerate, and whether the loss of hair cells can be prevented by a gene replacement approach. Long-term observational studies with morphological analysis of treated and untreated *Otof*^–/–^ organs of Corti will be required to predict a long-term outcome of gene therapy for DFNB9 patients.

## Data Availability Statement

The original contributions presented in the study are included in the article/supplementary material, further inquiries can be directed to the corresponding author/s.

## Ethics Statement

The animal study was reviewed and approved by Niedersächsisches Landesamt für Verbraucherschutz und Lebensmittelsicherheit (LAVES), Oldenburg, Germany.

## Author Contributions

NS and ER planned and supervised the research. US, AJF, and HA-M conducted the research and analyzed the data. All authors prepared figures. ER, NS, and US wrote the manuscript. All authors revised and approved the manuscript.

## Conflict of Interest

The authors declare that the research was conducted in the absence of any commercial or financial relationships that could be construed as a potential conflict of interest.

## Footnotes

1 http://imagej.net
